# Obesity and Hyperphagia With Increased Defective ACTH: A Novel *POMC* Variant

**DOI:** 10.1210/clinem/dgac342

**Published:** 2022-06-23

**Authors:** Eline S van der Valk, Lotte Kleinendorst, Patric J D Delhanty, Bibian van der Voorn, Jenny A Visser, M M van Haelst, Laura C G de Graaff, Martin Huisman, Anne White, Shosuke Ito, Kazumasa Wakamatsu, Yolanda B de Rijke, Erica L T van den Akker, Anand M Iyer, Elisabeth F C van Rossum

**Affiliations:** Obesity Centre CGG, Erasmus University Medical Center Rotterdam, 3000 CA Rotterdam, the Netherlands; Department of Internal Medicine, Division of Endocrinology, Erasmus University Medical Center Rotterdam, 3000 CA Rotterdam, the Netherlands; Obesity Centre CGG, Erasmus University Medical Center Rotterdam, 3000 CA Rotterdam, the Netherlands; Department of Clinical Genetics, Amsterdam UMC, University of Amsterdam, 1100 DD Amsterdam, the Netherlands; Obesity Centre CGG, Erasmus University Medical Center Rotterdam, 3000 CA Rotterdam, the Netherlands; Department of Internal Medicine, Division of Endocrinology, Erasmus University Medical Center Rotterdam, 3000 CA Rotterdam, the Netherlands; Obesity Centre CGG, Erasmus University Medical Center Rotterdam, 3000 CA Rotterdam, the Netherlands; Department of Internal Medicine, Division of Endocrinology, Erasmus University Medical Center Rotterdam, 3000 CA Rotterdam, the Netherlands; Department of Pediatrics, Erasmus University Medical Center, 3000 CA Rotterdam, the Netherlands; Obesity Centre CGG, Erasmus University Medical Center Rotterdam, 3000 CA Rotterdam, the Netherlands; Department of Internal Medicine, Division of Endocrinology, Erasmus University Medical Center Rotterdam, 3000 CA Rotterdam, the Netherlands; Department of Clinical Genetics, Amsterdam UMC, University of Amsterdam, 1100 DD Amsterdam, the Netherlands; Department of Internal Medicine, Division of Endocrinology, Erasmus University Medical Center Rotterdam, 3000 CA Rotterdam, the Netherlands; Department of Internal Medicine, Division of Endocrinology, Erasmus University Medical Center Rotterdam, 3000 CA Rotterdam, the Netherlands; Divison of Diabetes, Endocrinology & Gastroenterology, Faculty of Biology, Medicine and Health, University of Manchester, Manchester M13 9PL, UK; Institute for Melanin Chemistry, Fujita Health University, Toyoake, 470-1192, Japan; Institute for Melanin Chemistry, Fujita Health University, Toyoake, 470-1192, Japan; Department of Clinical Chemistry, Erasmus University Medical Center Rotterdam, 3000 CA Rotterdam, the Netherlands; Obesity Centre CGG, Erasmus University Medical Center Rotterdam, 3000 CA Rotterdam, the Netherlands; Department of Pediatrics, Erasmus University Medical Center, 3000 CA Rotterdam, the Netherlands; Obesity Centre CGG, Erasmus University Medical Center Rotterdam, 3000 CA Rotterdam, the Netherlands; Department of Internal Medicine, Division of Endocrinology, Erasmus University Medical Center Rotterdam, 3000 CA Rotterdam, the Netherlands; Obesity Centre CGG, Erasmus University Medical Center Rotterdam, 3000 CA Rotterdam, the Netherlands; Department of Internal Medicine, Division of Endocrinology, Erasmus University Medical Center Rotterdam, 3000 CA Rotterdam, the Netherlands

**Keywords:** pro-opiomelanocortin, genetic obesity, adrenocorticotropic hormone, melanocortin receptor

## Abstract

**Objective:**

Patients with pro-opiomelanocortin (POMC) defects generally present with early-onset obesity, hyperphagia, hypopigmentation and adrenocorticotropin (ACTH) deficiency. Rodent models suggest that adequate cleavage of ACTH to α-melanocortin–stimulating hormone (α-MSH) and desacetyl-α-melanocortin–stimulating hormone (d-α-MSH) by prohormone convertase 2 at the KKRR region is required for regulating food intake and energy balance.

**Methods:**

We present 2 sisters with a novel *POMC* gene variant, leading to an ACTH defect at the prohormone convertase 2 cleavage site, and performed functional studies of this variant.

**Results:**

The patients had obesity, hyperphagia and hypocortisolism, with markerly raised levels of ACTH but unaffected pigmentation. Their ACTH has reduced potency to stimulate the melanocortin (MC) 2 receptor, explaining their hypocortisolism.

**Conclusion:**

The hyperphagia and obesity support evidence that adequate cleavage of ACTH to α-MSH and d-α-MSH is also required in humans for feeding control.

A genetic diagnosis for obesity can be established in an estimated 2% to 9% of patients with severe obesity (1, 2). Since the first report in 1998, variants of the pro-opiomelanocortin (*POMC*) gene (MIM [609734]) have been found several times as a cause for human obesity (3, 4). Biallelic variants in the *POMC* gene can cause severe early-onset obesity, hyperphagia, red hair, and hypopigmentation, and a deficiency in adrenocorticotropin (ACTH) (3, 4). POMC is a precursor protein that is sequentially processed to generate ACTH, among other peptide hormones. ACTH is the sole ligand for the melanocortin-2 receptor/melanocortin-2 receptor accessory protein (MC2R/MRAP) receptor complex (5).

However, it also binds to the other melanocortin receptors; the MC1R, responsible for the regulation of pigmentation in the skin and hair follicles, and the MC3 and MC4 receptors, regulating energy intake and expenditure. ACTH is subsequently cleaved by prohormone convertase 2 (PC2), at tandem dibasic amino acid residues (KKRR) to produce ACTH_1-17_ and corticotropin-like intermediate lobe peptide (CLIP; Fig. 1). ACTH_1-17_ is further processed to produce its unacetylated form desacetyl-α-MSH (d-α-MSH) and then α-melanocyte–stimulating hormone (α-MSH), which has been shown to be required for the regulation of food intake and energy balance (6-8). Our case individuals presented with a phenotype of an unusual combination of hypocortisolism with high ACTH, early-onset obesity and hyperphagia, and unaffected pigmentation. Their mutant ACTH^KRR^ has considerably reduced potency at the ACTH receptor (MC2R), explaining their hypocortisolism. Moreover, this is the first report of a human variant in the PC2 cleavage site. Inefficient processing of ACTH to α-MSH at this site could explain not only the high plasma levels of ACTH but also the hyperphagia and early-onset obesity in these patients.

**Figure 1.**
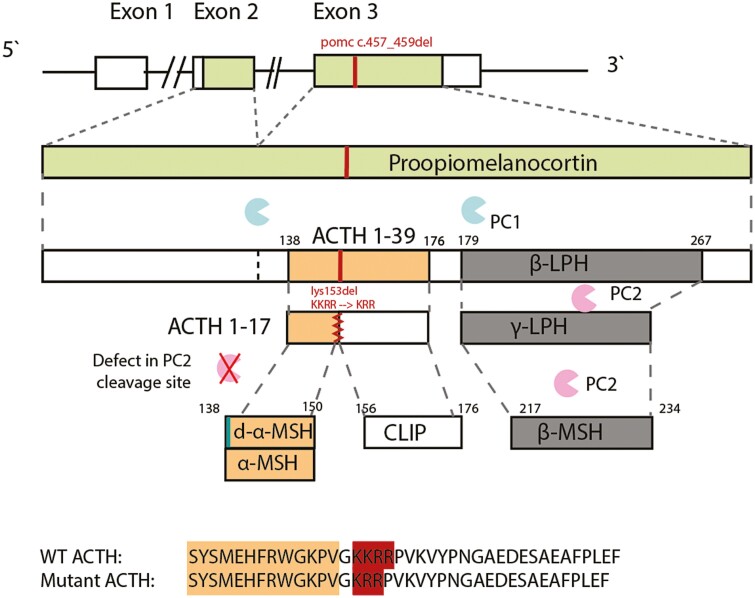
The POMC variant and plausible protein defect. Pro-opiomelanocortin (POMC) is sequentially cleaved to adrenocorticotropin (ACTH) 1-39 by prohormone convertase (PC) 1 and subsequently to ACTH 1-17 by PC2. Carboxypeptidase E (CPE) then processes ACTH 1-17 to desacetyl-α-melanocortin–stimulating hormone (MSH) and α-MSH. The amino acid sequence alignment is shown for wild-type (WT) ACTH and for the mutant ACTH (the amino acid sequence of α-MSH is highlighted in orange, and the PC2 cleavage site in red). PC2 cleaves β-lipotropic hormone (β-LPH) to γ-LPH and β-endorphin. β-LPH is further cleaved to β-MSH (this region is unaffected by the *POMC* variant).

## Clinical History

The female index patient (patient A, current age 27 years, body mass index 41.59) was seen at age 7 years at the department of pediatric endocrinology because of the suspicion of adrenal insufficiency. Since age 1.5 years she had been overweight with overt hyperphagia, which soon turned to early-onset obesity. Since age 5 years she had been hospitalized multiple times for recurrent hypoglycemias, respiratory tract infections, and vomiting. At the time of the first evaluation at the outpatient clinic, she had recently been discharged from the pediatric intensive care unit where she was treated for respiratory insufficiency and septic shock, and the diagnosis of severe hypocortisolism was made (serum cortisol < 28 nmol/L). There was a markedly raised plasma level of ACTH (87.2 pmol/L, upper limit of normal range 11 pmol/L), and hydrocortisone substitution therapy had been initiated. Further analysis showed no mineralocorticoid deficiency, no autoantibodies against the adrenal gland or antithyroid peroxidase antibodies, and adrenoleukodystrophy was excluded. A computed tomography scan revealed an anatomically normal left adrenal gland and no visible right adrenal gland. Stimulation with synthetic ACTH did not lead to a sufficient increase of cortisol levels. She had dark hair and normal skin pigmentation considering her South Asian background. At age 14 she developed overt primary hypothyroidism (free thyroxine 9.6 pmol/L, reference range, 12-26 pmol/L; thyrotropin 10.5 mU/L, reference range, 0.4-4.3 mU/L), which was supplemented, and later, at age 18, a growth hormone (GH) deficiency (insulin-like growth factor-1 levels 15.8 nmol/L, lower limit of normal 25 nmol/L; and insufficient increase of GH after stimulation with clonidine and arginine) without visible abnormalities in the pituitary on magnetic resonance scans, which was also supplemented. Her sister (patient B) presented at age 3 years with a similar clinical picture: primary hypocortisolism (serum cortisol < 28 nmol/L) with high plasma ACTH levels (1036 pmol/L), early-onset obesity (at age 3 her weight and height were > 2 SDs above the average, at age 16 her body mass index was 30.3) and hyperphagia, but also an autism spectrum disorder and mild cognitive impairment. Their parents were consanguineous (first cousins). Their brother died at age 19 of lymphomatoid granulomatosis. Neither their brother nor their parents had obesity or adrenal insufficiency.

### Genetic Testing

**Figure 2.**
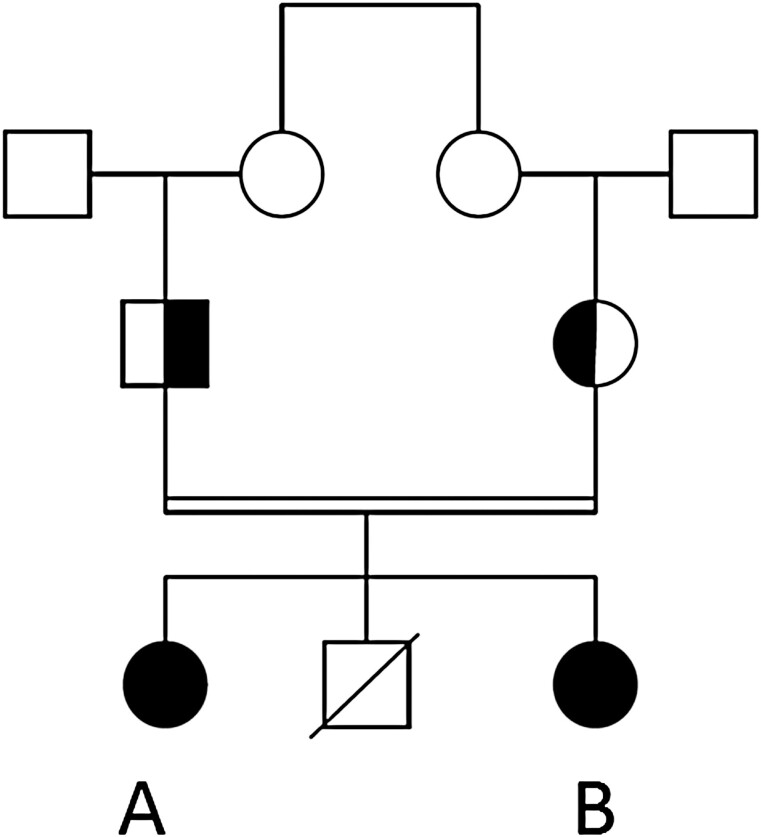
Family tree of the participants. Individuals A and B have a novel, homozygous pro-opiomelanocortin mutation (marked in black). Their parents are heterozygous carriers.

Between 2002 and 2009, genetic testing was performed for ACTH receptor resistance syndromes, revealing no abnormalities in *NR0B1*, *MC2R*, *GNAS*, and *MRAP*. In 2018, whole-exome sequencing filtered on the regions of homozygosity showed a novel homozygous in-frame deletion in the *POMC* gene that has never been reported to date (c.457_459del, p.[Lys153del]; NM_001319204.1). This homozygous *POMC* variant was also identified in the younger sister via Sanger sequencing. The parents are both heterozygous carriers (family tree depicted in Fig. 2). We found no other genetic obesity disorders (virtual gene panel assessed on the diagnostic whole-exome sequencing data included the following genes: *ALMS1*, *BDNF*, *CPE*, *GNAS*, *LEP*, *LEPR*, *MAGEL2*, *MC3R*, *MC4R*, *PCSK1*, *PHF5*, *SH2B1*, *SIM1*, and *VPS13B*).

### Follow-up

Unfortunately, shortly after the results of the whole-exome sequencing of patient A were obtained, patient B was diagnosed with severe primary hemophagocytic lymphohistiocytosis in the presence of a pathogenic homozygous *PRF1* variant, ultimately resulting in her death, despite intensive chemotherapy treatment. We were unable to obtain serum from her before the initiation of her treatment, which included high doses of corticosteroids, and her relatives did not consent to autopsy. During the course of this illness, shortly before she died, she also developed a mild central hypothyroidism. However, it was unclear if this was due to the critical illness or part of the clinical picture of the *POMC* mutation. Postmortem analysis of tissue from their deceased brother demonstrated that he was a carrier of the *PRF1* variant but not of the *POMC* variant. The homozygous *PRF1* variant, together with the expected functional defects in the natural killer cells, was also found in patient A, for which she received an allogenous stem cell transplantation. Once patient A has reached a stable clinical condition, after stem cell transplantation, treatment with the MC4R agonist setmelanotide may be considered as a therapeutic option to treat her hyperphagia and obesity (9).

### Functional Testing

#### Contribution of adrenocorticotropin precursors to ACTH levels

ACTH precursors (POMC and pro-ACTH) were determined in the plasma of patient A and their mother, using a specific immunoassay as previously described (10).

#### Potency of mutant adrenocorticotropin to stimulate the melanocortin 2 receptor in vitro

Wild-type (WT) (ACTH^KKRR^, peptide 1-39) and mutant ACTH^KRR^ peptides were custom synthesized by Biomatik. To evaluate the potency of the ACTH^KRR^ on the MC2R, HEK293 cells stably transfected with MC2R and MRAP expression plasmids were used, as described previously (11). The cells were stimulated with increasing doses of ACTH^KKRR^ or ACTH^KRR^ peptides for 6 hours. Cyclic adenosine 5′-monophosphate (cAMP) levels were assessed using a luciferase reporter assay, as described previously (12). The half-maximal effective concentration (EC_50_) was calculated both for mutant and WT ACTH. Similarly, the potency of ACTH^KRR^ was evaluated on MC4R-expressing cell lines (complementary DNA clone obtained from cDNA Resource Center; www.cdnd.org).

#### Hair pigmentation analysis

The melanin determination of hair samples of the 2 patients and their mother were performed as described elsewhere (13-15).

## Results

### Adrenocorticotropin Precursors Do Not Contribute to Measured Plasma ACTH Levels

Plasma levels of ACTH precursors POMC and pro-ACTH as determined using a specific immunoassay were 88 pmol/L in patient A (normal range, 0-40 pmol/L), in the presence of ACTH levels of 225 pmol/L. Since ACTH precursors cross-react at around 2% in most ACTH assays, at this concentration, it is unlikely that they contributed substantially to the total ACTH measurement.

### Mutant Adrenocorticotropin^KRR^ Shows Reduced Potency to Stimulate Melanocortin 2 Receptor

**Figure 3.**
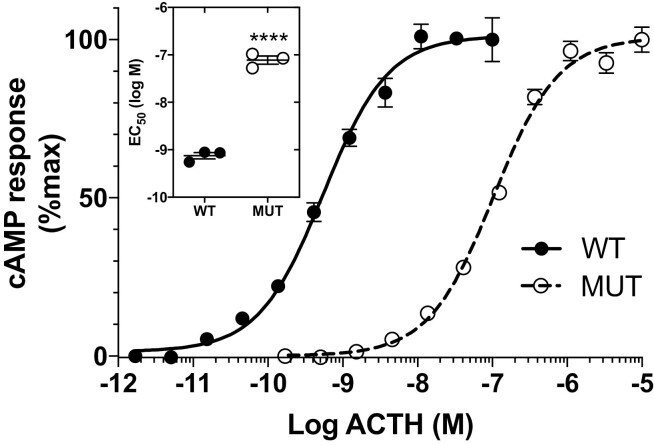
Mutant adrenocorticotropin (ACTH)^KRR^ (MUT) is less potent than wild-type ACTH^KKRR^ (WT) in stimulating MC2R. Cyclic adenosine 5′-monophosphate (cAMP) response in MC2R-MRAP HEK-293 cells stimulated with ACTH^KRR^ and ACTH^KKRR^ (n = 3, doses in triplicate. Difference in half-maximal effective concentration (EC_50_); ****P < .0001).

**Figure 4.**
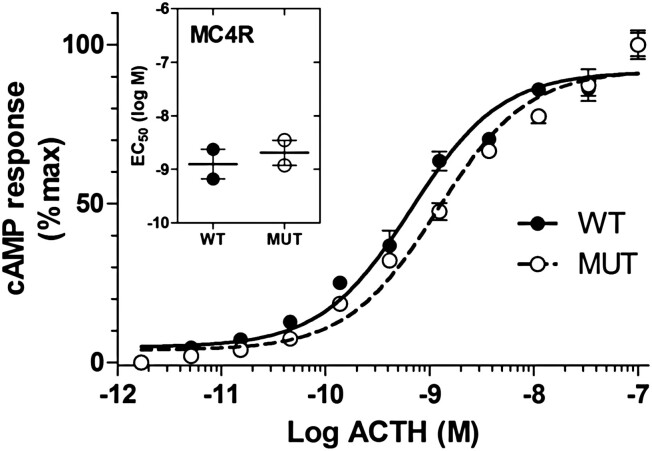
Mutant adrenocorticotropin (ACTH)^KRR^ (MUT) has similar potency compared to wild-type ACTH^KKRR^ (WT) in stimulating MC4R. Cyclic adenosine 5′-monophosphate (cAMP) response in MC4R-transfected HEK-293 cells stimulated with ACTH^KRR^ and ACTH^KKRR^ (peptide doses in triplicate, 2 independent experiments. Difference in half-maximal effective concentration [EC_50_]).

Mutant ACTH^KRR^ elicited an approximately 100-fold lower cAMP response as compared to WT ACTH^KKRR^ in HEK293 cells stably transfected with MC2R/MRAP. The mean EC_50_ concentrations of mutant (80.4 ± 15 nM) and WT (0.8 ± 0.1 nM) ACTH were significantly different (*P* < .0001; *t* test) (Fig. 3). There was no difference in potency between ACTH^KKRR^ and ACTH^KRR^ at the MC4R (Fig. 4).

### Hair Pigmentation Is Not Affected by Pro-Opiomelanocortin Variant

Hair samples of the 2 patients and their mother were eumelanic (> 90% eumelanin) and were not different from each other (Supplementary Data S1) (16)).

## Discussion

We present a unique case of 2 siblings exhibiting primary hypocortisolism with high levels of ACTH, obesity, and hyperphagia, in the presence of a novel homozygous *POMC* variant located within the PC2 cleavage site. Collectively, this results in a clinical picture with 1) normal hair pigmentation, reflecting unaffected activation of the MC1R in hair follicles; 2) hypocortisolism with high ACTH, corresponding to a reduced in vitro potency of ACTH^KRR^ at MC2R/MRAP; and 3) obesity and hyperphagia, which are likely explained by the lack of α-MSH and desacetyl-α-MSH to stimulate the MC4R in the hypothalamus, despite normal potency of ACTH at the MC4R and no likely abnormalities in β-melanocortin–stimulating hormone (β-MSH).

Patients with a POMC deficiency generally have hypocortisolism with low or undetectable plasma ACTH levels (Table 1 provides a summary of classic endocrine findings in patients with a POMC deficiency) (4). There is one previous report describing 2 unrelated hypocortisolemic patients with POMC variants who had uncharacteristically high plasma ACTH levels (17). This variant (POMC p. R145C, equivalent to ACTH p. R8C and α-MSH p. R8C) occurs in the amino acid sequence HFRW, which is essential for ACTH and α-MSH to activate the MC receptors. Their phenotype included, besides glucocorticoid deficiency, also red hair and obesity (17). In contrast, our patients did not display hypopigmentation or red hair, and their POMC variant is located in the KKRRP motif, which is the other site that is required for specific binding of ACTH to MC2R (7, 11). We found that the variant in our patients resulted in an approximately 100-fold lower potency of ACTH to stimulate MC2R/MRAP, explaining their hypocortisolism. Whereas initially the high ACTH led to the suspicion of primary adrenal insufficiency, the reduced potency of ACTH at the MC2R/MRAP shows that there is actually a secondary adrenal insufficiency. Theoretically, it is possible that the high ACTH observed in the serum of our patients could be explained by cross-reactivity of the ACTH assay with ACTH precursors such as POMC and pro-ACTH. However, the relatively low serum levels of ACTH precursors estimated in a specific assay (10) suggest that their contribution to the measured serum ACTH level is negligible.

**Table 1.** Comparison of symptoms of pro-opiomelanocortin deficiencies between our patients vs patients reported in the literature

Classical clinical symptom	Involved melanocortin receptor	Relevant agonists derived from POMC (6)	Main function	Clinical findings of other *POMC* cases in literature (21)	Clinical picture of patients A and B	Suspected mechanism in patients A and B
Hypopigmentation and red hair	MC1R	ACTH, α-MSH, β-MSH, γ-MSH	Stimulates pigmentation	Red hair in 50% of patients, reddish brown in 30%	Black hair, unaffected pigmentation	Normal pigmentation, samples not different from mother (heterozygous carrier), probably due to intact HFRW motif and sufficient other contributors to pigmentation in people of color individuals
Adrenal insufficiency	MC2R	ACTH	Stimulates production and secretion of glucocorticoids from adrenal cortex	Secondary adrenal insufficiency with low/undetectable levels of ACTH (with the exception of 2 cases with high levels of bioinactive ACTH (16))	Adrenal insufficiency with high levels of mutant ACTH^KKR^	ACTH^KKR^ has 100-fold lower potency at MC2R/MRAP
Obesity and hyperphagia	MC4R	ACTH, α-MSH, d-α-MSH (7), β-MSH, γ-MSH	MC4R signaling reduces appetite and energy expenditure	Severe, early-onset obesity with hyperphagia	Severe, early-onset obesity with hyperphagia	Impaired MC4R signaling due to absence of α-MSH and d-α-MSH (despite normal potency of ACTH at MC4R) (7)
Central hypothyroidism	MC4R	α-MSH (22)	Stimulation of thyroid axis	Central hypothyroidism in 36% of cases	Central hypothyroidism developed in adolescence (case A) and possible central hypothyroidism (patient B)	Absence of α-MSH and d-α-MSH. Normal potency of ACTH at MC4R
GH deficiency	Unclear	Unclear	Unclear	GH deficiency in 14% of reported patients, all in adolescence. Accelerated growth in infancy	Accelerated growth in childhood, in adolescence GH deficiency (patient A).	Unclear

Abbreviations: α-MSH, α-melanocortin–stimulating hormone; ACTH, adrenocorticotropin; d-α-MSH, desacetyl-α-melanocortin–stimulating hormone; GH, growth hormone; MC1R, melanocortin 1 receptor; MC2R, melanocortin 2 receptor; MC4R, melanocortin 4 receptor; POMC, pro-opiomelanocortin.

Early-onset severe obesity with hyperphagia is a hallmark phenotype associated with defective POMC, following the lack of POMC processing to α-MSH, d-α-MSH, and β-MSH, the most important ligands of the MC4R in the hypothalamus (6-8). Numerous studies have elucidated the role of the MC4R pathway and, to a lesser extent, the MC3R in the regulation of food intake and energy expenditure, with impaired signaling through these receptors being associated with obesity (6). We hypothesize that in our patients the KKR mutation, located in the PC2 recognition site in ACTH, impairs cleavage to the main ligands of the MC4R, α-MSH, and d-α-MSH (7). Indeed, mice with a similar variant in the same region (QKQR) had impaired processing to d-α-MSH and α-MSH, and had a phenotype of melanocortin obesity (7). A recent study involving human hypothalamic neuronal cell lines and postmortem human brains demonstrated that d-α-MSH and β-MSH (originating from a different part of the POMC protein), are the most abundant MC4R ligands in the human hypothalamus (8), regulating eating behavior and energy balance. Because there was no permission for autopsy of patient B, we could not prove the absence of α-MSH and d-α-MSH in the hypothalamus. However, in our case patients, the location of this *POMC* variant, together with the high ACTH levels, strongly suggests that their obesity and hyperphagia were indeed the consequence of impaired PC2 cleavage and thereby impaired activation of MC4R.

The clinical picture of our patients can thus be considered an additional affirmation of insights from animal models, supporting the concept that cleavage of ACTH to α-MSH and d-α-MSH is also crucial for regulating energy balance in humans, despite normal potency of ACTH at the MC4R and unaffected β-MSH (7, 8).

Although α-MSH and d-α-MSH are potent in vitro ligands to the MC1R (18, 19), their presence is not required for hair pigmentation. This is likely maintained by other factors such as ACTH, β-MSH, γ-MSH, and β-endorphin (6, 20) or by constitutive activity of MC1R (21).

Lastly, these patients presented with GH deficiency and central hypothyroidism, features that are known from other cases of *POMC* deficiency (22). It has been postulated that this is due to the absence of MC4R stimulation (which indirectly stimulates thyrotropin-releasing hormone). The presence of central hypothyroidism in these cases can thus be considered an additional strong argument for the concept that there is indeed no or impaired cleavage of ACTH to α-MSH and d-α-MSH, and that they indeed do not activate the MC4R (23).

GH deficiencies have also been reported previously in the context of *POMC* mutations, but seem to occur mainly in adolescence, as in our patients (22). The exact pathophysiology behind this to our knowledge has not yet been elucidated.

To summarize, this is the first report of patients with a variant in the KKRRP motif of the *POMC* gene, who had high ACTH levels, hyperphagia, and obesity. The KKRRP motif is important for the binding of ACTH to MC2R, explaining their hypocortisolism, but also involves loss of the PC2 cleavage site, likely causing obesity and hyperphagia through the absence of α-MSH and d-α-MSH. These sisters are the first human examples with a unique, and to our knowledge not yet reported, clinical picture, demonstrating that the KKRRP motif of ACTH is critical for feeding control.

## Data Availability

Data sharing is not applicable to this article because no data sets were generated or analyzed during the present study.

## Abbreviations

α-MSH: α-melanocortin–stimulating hormone
ACTH: adrenocorticotropin
cAMP: cyclic adenosine 5′-monophosphate
d-α-MSH: desacetyl-α-melanocortin–stimulating hormone
EC_50_: half-maximal effective concentration
GH: growth hormone
MC1R: melanocortin 1 receptor
MC2R: melanocortin 2 receptor
MC4R: melanocortin 4 receptor
MRAP: melanocortin-2 receptor accessory protein
PC2: prohormone convertase 2
POMC: pro-opiomelanocortin
WT: wild-type
